# Rare bird forecast: A combined approach using a long‐term dataset of an Arctic seabird and a numerical weather prediction model

**DOI:** 10.1002/ece3.11388

**Published:** 2024-06-25

**Authors:** Masayuki Senzaki, Kenta Tamura, Yoshiaki Watanabe, Megumi Watanabe, Tomonori Sato

**Affiliations:** ^1^ Faculty of Environmental Earth Science Hokkaido University Sapporo Hokkaido Japan; ^2^ East Hokkaido Bat Research Institute Ohzora Hokkaido Japan

**Keywords:** animal forecast, bird watching, ecological climatology, ecosystem services, numerical weather prediction model, rare species

## Abstract

Wildlife observation is a popular activity, and sightings of rare or difficult‐to‐find animals are often highly desired. However, predicting the sighting probabilities of these animals is a challenge for many observers, and it may only be possible by limited experts with intimate knowledge and skills. To tackle this difficulty, we developed user‐friendly forecast systems of the daily observation probabilities of a rare Arctic seabird (Ross's Gull *Rhodostethia rosea*) in a coastal area in northern Japan. Using a dataset gathered during 16 successive winters, we applied a machine learning technique of self‐organizing maps and explored how days with gull sightings were related to the meteorological pressure patterns over the Sea of Okhotsk (Method A). We also built a regression model that explains the relationship between gull sightings and local‐scale environmental factors (Method B). We then applied these methods with the operational global numerical weather prediction model (a computer simulation application about the fluid dynamics of Earth's atmosphere) to forecast the daily observation probabilities of our target. Method A demonstrated a strong dependence of gull sightings on the 16 representative weather patterns and forecasted stepwise observation probabilities ranging from 0% to 85.7%. Method B also showed that the strength of the northerly wind and the advancement of the season explained gull sightings and forecasted continuous observation probabilities ranging from 0% to 95.5%. Applying these two methods with the operational global numerical weather prediction model successfully forecasted the varied observation probabilities of Ross's Gull from 1 to 5 days ahead from November to February. A 2‐year follow‐up observation also validated both forecast systems to be effective for successful observation, especially when both systems forecasted higher observation probabilities. The developed forecast systems would therefore allow cost‐effective animal observation and may facilitate a better experience for a variety of wildlife observers.

## Graphical abstract

Wildlife observation is a popular recreation worldwide, and sightings of rare animals are often highly desired. We developed user‐friendly forecast systems of the daily observation probabilities of a rare seabird species (Ross's Gull *Rhodostethia rosea*) in a coastal area in northern Japan. The developed forecast systems are useful for the cost‐effective observation of this beautiful bird.

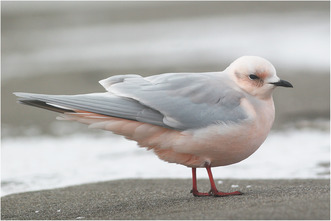

## 1 INTRODUCTION

Wild animal observation is a popular recreation worldwide (Buckley, 2011). For instance, birdwatching and bird photography are reported to be one of the fastest growing recreational activities over the last three decades from 1982 to 2009 (Michel et al., 2020). A key to making animal observation successful is meeting the personal desire to observe target animals (Davis et al., 1997; Mutanga et al., 2017). Although unpredictability may also be an exciting factor of animal observation, knowing/increasing the observation likelihood may be important to achieve this. It is, therefore, crucial to develop a reliable and user‐friendly system that forecasts the observation likelihood of targets for the decision‐making of a variety of observers with diverse attributes.

Both locally and globally rare animals represent typical targets for wildlife recreation (Mariyam et al., 2022; Senzaki et al., 2017; Veríssimo et al., 2009). Especially popular are avian vagrants or rare migrants that are found outside their usual distribution ranges (Callaghan et al., 2018, 2020). Both internal and endogenous factors can affect avian vagrancy or a forced landing in avian migrants (Lees & Gilroy, 2022). For example, previous studies have revealed that its likelihood may increase under specific meteorological conditions, such as the timings of wind drift, squalls, and cyclone passages, and the developed low‐pressure systems (Gilroy & Lees, 2003; Lees & Gilroy, 2009). Therefore, we may be able to know their daily observation probabilities by explicitly incorporating the meteorological mechanisms of avian vagrancy/migration into the numerical weather prediction models used for weather forecasts (i.e., a computer simulation application about the fluid dynamics of Earth's atmosphere), which can represent various meteorological phenomena based on atmospheric dynamics and physical processes at small to large spatial–temporal scales. The application of the numerical weather prediction model or weather forecasts in ecological studies has mostly been limited to those at the community to ecosystem levels (e.g., Gutman & Ignatov, 1998; Van Doren & Horton, 2018) but its more flexible application would have great potential not only to facilitate our understanding of the previously less‐understood mechanisms about rare bird vagrancy/migration and their population trends but also to advance familiarity of environmental science to the general public.

**FIGURE 1:**
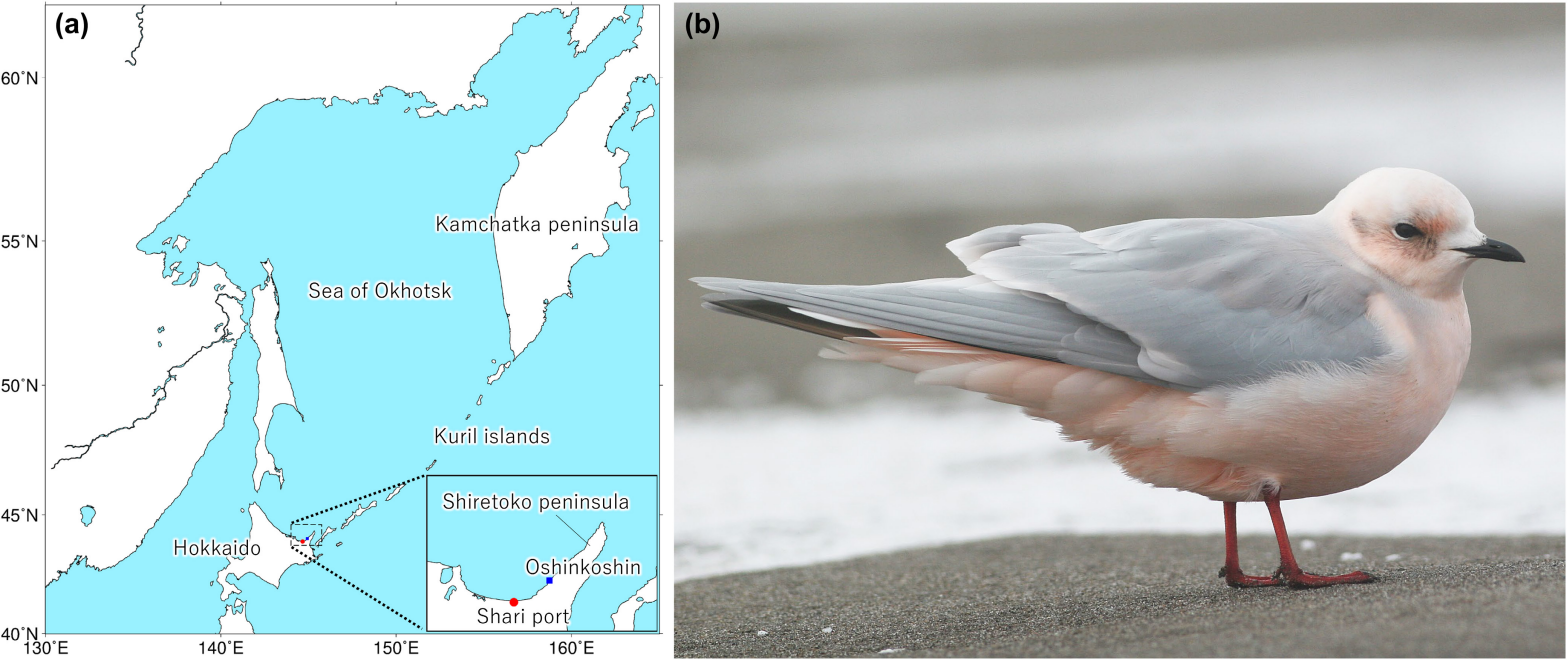
(a) Study sites (Shari port and Oshinkoshin in the Shiretoko Peninsula) and major geographical regions around the Sea of Okhotsk. (b) An adult Ross's Gull observed on a coastline of the Shiretoko Peninsula. Photographed by Hiraku Senzaki.

Here, we aim to develop real‐time forecast systems for the vagrancy/migration events of a rare avian species using a numerical weather prediction model. To do so, we focus on a long‐term monitoring dataset of Ross's Gull *Rhodostethia rosea* in northern Japan (Figure 1). This species breeds in the Arctic tundra in east Eurasia to North America and is assumed to winter offshore at the edge of the Arctic pack ice (Béchet et al., 2000; Egevang & Boertmann, 2008; Maftei et al., 2012, 2015). However, wandering individuals are known to occasionally be recorded during winter in the south to the sub‐Arctic and/or temperate zones across the Northern Hemisphere (Bledsoe & Sibley, 1985). Because of its iconic appearance, Ross's Gull is one of the most aspirational targets for birdwatchers across these regions.

**FIGURE 2:**
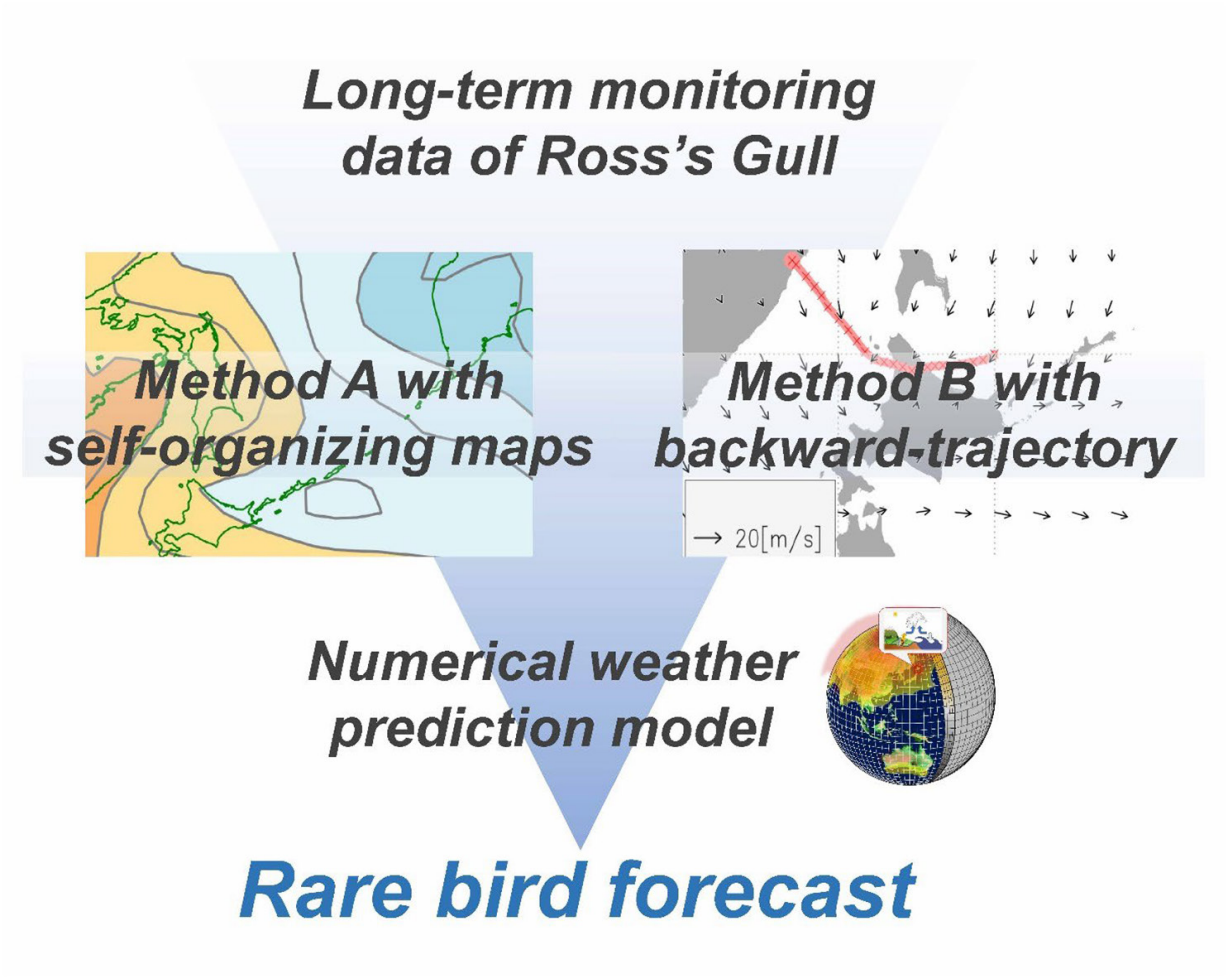
Flowchart of the study.

The Shiretoko Peninsula along the Sea of Okhotsk in northern Japan (Figure 1) is one of the world's most reliable observation spots for non‐breeding Ross's Gull (Mori, 1976; Nakagawa, 1987). In addition to the known preferences of pelagic habitats and the edges of sea ice extent in this species (Maftei et al., 2015), anecdotal documentation has suggested that records of this species around the peninsula are likely related to some specific meteorological factors such as strong winds during winter, especially those from a north direction, and the extent of sea ice coverage of the Sea of Okhotsk (Mori, 1976, Nakagawa, 1987). Local birdwatchers have monitored the occurrences of this species in various climates for the past 16 years between 2004 and 2020. Using this long‐term dataset, we first explored the relationship between the days with gull sightings and the pressure patterns around the Sea of Okhotsk (Method A, Figure 2). We also constructed a statistical model that explicitly explains gull sightings based on the aforementioned key environmental factors, such as wind speed, wind directions, and sea ice coverage of the Sea of Okhotsk (Method B, Figure 2). We finally developed two different real‐time forecast systems for Ross's Gull observation by incorporating these two methods into a numerical weather prediction model (Figure 2). Method A would be more cost‐effective when sufficient data were obtained under various meteorological conditions and may provide a more reliable forecast probability if the gull observations were dependent on large‐scale atmospheric pressure patterns. Method B would be more reliable when the gull observation was tightly linked to specialized climatic factors more than general atmospheric pressure patterns.

## 2 MATERIALS AND METHODS

### 2.1 Field monitoring and dataset arrangement

Two of the authors (Y. Watanabe & M. Watanabe) led the seabird count surveys on a total of 1454 occasions at multiple locations along the coastline at Monbetsu city, Abashiri city, Koshimizu town, and Shari town from May 2004 to August 2020 (Watanabe & Watanabe, unpublished; Watanabe, 2021). Using a 10× binocular and 20–60× telescope, surveyors counted the abundance of each identified species within 2–3 km from the observatories with clear visibility. Because our primary surveyors are amateur researchers, those counts were conducted when surveyors were off from their job, but long‐term efforts covered various days under varied climatic conditions. Each count was continued for a minimum of 15–30 min (Watanabe, 2021) but was extended for several hours, and sometimes into the sunset when climatic conditions seemed suitable for Ross's Gull southward migration and/or remarkable seabird movement/migration occurred (see also the Method A and Method B sections for how to control different observation efforts). From this original dataset, we used 581 observation data obtained at two locations (Shari port and Oshinkoshin observatory) along the Shiretoko Peninsula where Ross's Gull has most frequently been recorded. We then further limited to 322 independent data from November to February (i.e., 60 presence and 262 absence observation occasions) because 100% of Ross's Gull records have been obtained in those periods. Moreover, our primary purpose was to understand/forecast the climatic conditions of the initial timing when Ross's Gull arrived in the study area. However, the dataset included some data obtained on several days after its initial arrival (i.e., successive days with the same individuals/flocks observed). We therefore confined those data as follows. For days with gull sightings (i.e., presence days), when the gull was sighted at <2‐day intervals (e.g., on successive days or those at a 1‐day interval), we considered those periods to have the same wandering individual(s) at each location and used the highest count day(s) from the periods. We removed the remaining presence days of the same periods from the dataset. This assumption was supported by opportunistic, but multiple observations of the same individuals based on unique plumages. When we had more than two gull presence days with the highest counts in each period, we used the first highest count day only. All the other presence days were kept for the following analysis. For days without gull sightings (i.e., absence days), we removed absence days within 2 days from a day with gull sightings. When the other absence days were obtained at <2‐day intervals, we only kept the first absence days. The other absence days were retained. As a result, we used the final dataset with 178 unique‐day records (i.e., 33 presence and 145 absence days) between 11th November 2004 and 10th February 2020 for the following analyses. The average observation time per day (±SD) for this dataset was 77.47 ± 65.83 min.

### 2.2 Developing forecast systems

This study proposes two forecast methods (Figure 2). Method A is based on analyzing the large‐scale surface pressure pattern in the study area. Method B analyzes the airmass that is assumed to travel together with the gull and estimates the pathway of the gull's movement. Both methods built the statistical relationship between meteorological conditions and in situ monitoring of the target species. We next performed the daily forecast of the gull observation probabilities using forecasted meteorological fields as an input of both methods. The forecast data were obtained from the operational numerical weather prediction model. It means the forecasted data are produced by computer simulation about the fluid dynamics of Earth's atmosphere, and the simulation uses a software package called numerical weather prediction model. Operational refers to forecasts produced by an institution (typically a government agency but sometimes a private company) providing an ongoing and supported time‐critical service (Pagano et al., 2024). The forecast data are identical to those used by the Japan Meteorological Agency (JMA) for daily weather forecasts. Based on both methods, gull observation probabilities were forecasted for 1–5 days ahead of the focal days. The forecast duration (i.e., 1–5 days ahead) was determined based on the data availability of the operational numerical weather prediction model.

#### 2.2.1 Method A: Forecast based on SLP patterns

Northwesterly winds blow predominantly over Hokkaido during the winter season. This is driven by the strong surface pressure gradient between the Siberian High to the west and the Aleutian Low to the east of Hokkaido. According to our preliminary investigation, the strong northerly wind appeared to be associated with the observation of the focal species in the study area. This suggests that such an event can be predicted by identifying the surface pressure patterns that drive winds favorable to the gull vagrancy/migration. Method A demonstrates the probabilistic forecast of the focal species migration using the forecasts of surface pressure patterns created by a numerical weather prediction model in addition to the probability of migration determined prior for each typical type of pressure pattern.

##### Self‐organizing maps

We classified the daily surface pressure patterns during the winter around the Sea of Okhotsk (35–65° N, 135–165° E) into some representative types. The technique of Self‐Organizing Maps (SOMs; Kohonen, 1982), a machine learning method, was used for the classification. The classification by SOMs considers similarities among high‐dimensional input datasets and displays the results as a two‐dimensional map. The SOMs method has been widely utilized in the fields of science and technology and is often used not only for the classification of surface pressure patterns in meteorology (e.g., Ohba et al., 2015; Tamura & Sato, 2022) but also for understanding the relationship between species occupancy and multi‐dimensional habitat parameters in ecology (Heim et al., 2023).

##### Data and weather pattern categorization

**FIGURE 3:**
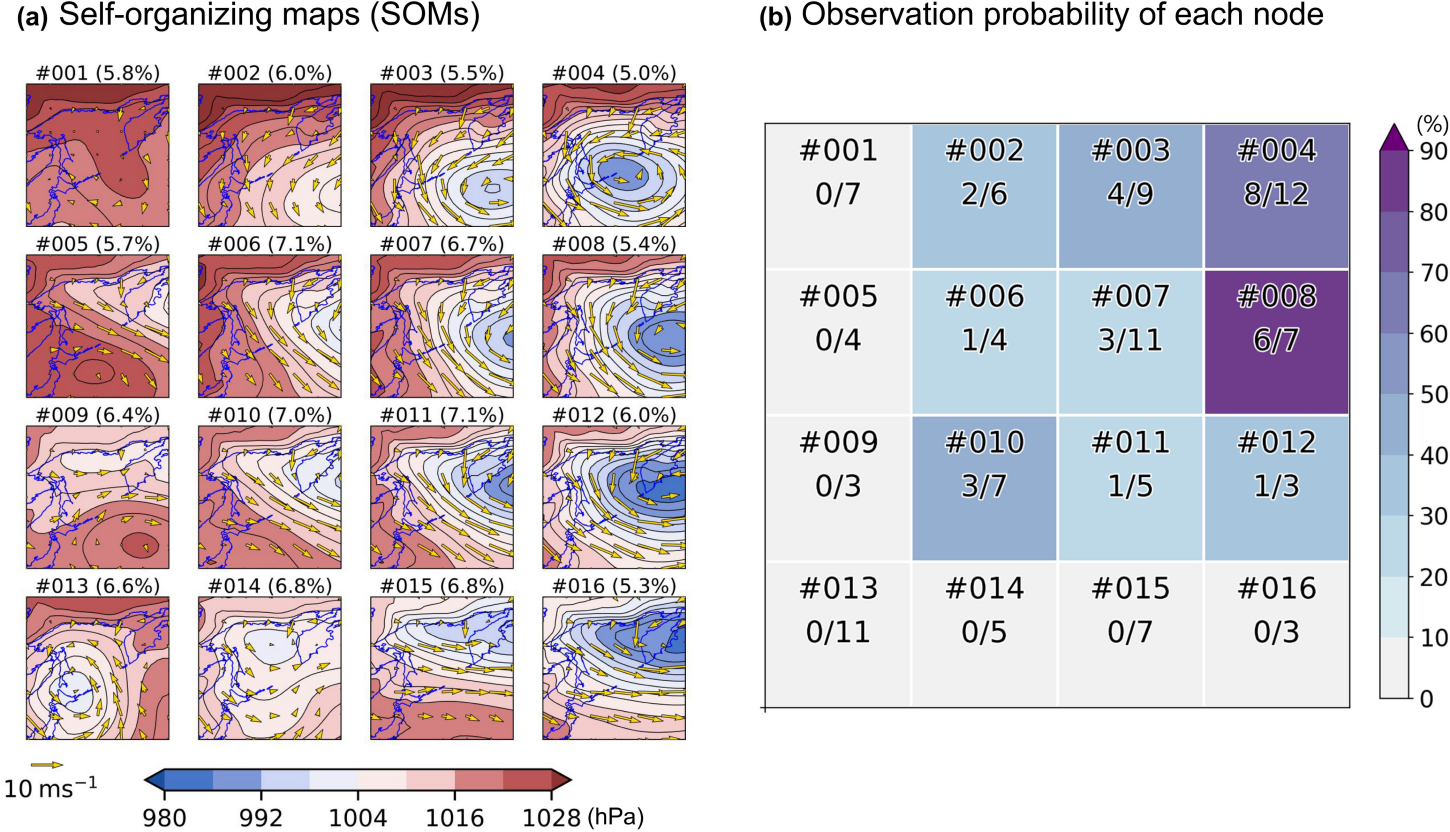
Self‐organizing maps (SOMs) to forecast the gull observation of the Shiretoko Peninsula. (a) A total of 16 nodes classified with this technique. The node IDs are indicated with #001, 002, …, and #016. Values in each parenthesis indicate the frequency of each node during winter (i.e., November–March); for example, it indicates that the pressure pattern of #001 occurs at 5.8% within this timeframe. (b) The observation frequency and probability of each node; for example, Ross's Gull was observed in 8 of 12 cases for node #004.

We first created 16 weather patterns that represent typical sea level pressure (SLP) distributions during the winter season using the SOM technique (Figure 3). The input data for this step were daily SLP patterns during November through March over 1981/1982–2019/2020 winters (i.e., 38 winters and 5 months = 5748 days) obtained from the Japanese 55‐year Reanalysis dataset (JRA‐55) (Kobayashi et al., 2015). By inputting the SLP data from 5748 days into the SOM algorithm, we classified the SLP patterns into 16 types (Figure 3). Each type shows the averages of the SLP and surface wind distributions of the corresponding pattern (Figure 3). The 16 SLP patterns were displayed by 4 × 4 alignment, and each fragment is referred to as a “node.” It is interpreted that the location of the low‐pressure system is a key factor in characterizing the winter SLP pattern. For instance, the upper right nodes in Figure 3a represent the low pressure to the east of Hokkaido, and the lower right nodes represent the low pressure located near the Kamchatka Peninsula. These characteristics are also consistent with the characteristics of the winter climate in this region; low‐pressure systems generated over the Eurasian continent or the Sea of Japan frequently pass around Hokkaido (Chen et al., 1991). We next estimated the gull observation probability for each node. To account for the effects of different observation durations among the observation days on the detection probability of Ross's Gull, we checked how many minutes were required to detect the first Ross's Gull of the day for 39 of 60 presence days of the raw dataset. This showed that the first detection happened within 14.48 ± 28.33 min, indicating observation efforts with >45 min can ensure the target detection of the day. For Method A, we therefore removed 74 days when observation was conducted for <45 min from the final dataset. The SLP pattern at each of 104 observation days (29 sighted and 104 unsighted days) belongs to a certain node, which most resembles the SLP pattern, out of the 16 nodes (Figure 3a). The result of this classification is presented as the denominator in each node (Figure 3a). In the same manner, the numerator in each node indicates the number of sighted days. As a result, the shading in Figure 3 denotes the probability based on the in situ monitoring. In the forecast method, the probability value at the node having the most similar SLP pattern to the predicted SLP pattern is adopted.

#### 2.2.2 Method B: Forecast based on a statistical model and backward trajectory

This method involved building a statistical model relating gull occurrence to specific meteorological factors. Hence, we constructed a generalized linear model (GLM) with a binomial error on the final dataset. We used the presence/absence of the gull as the response variable. For explanatory variables, we used (1) the ordinal date (1 = November 1st), (2) the proportion of sea ice surface of the Sea of Okhotsk (hereafter “sea ice extent”), (3) the latitudes of the air parcel 24, 48, and 72 h before it arrived at the observation site (hereafter “air parcel latitude”), and (4) the direction/angle of the air parcel's location 24, 48, and 72 h before it arrived at the observation site, defined as degrees clockwise from north (hereafter “air parcel angle”). We also included the observation duration (minutes) of the day as an additional explanatory variable to account for varied observation efforts. For the sea ice extent, we used the daily data of the sea ice fraction from OISST (Huang et al., 2021). We calculated the sea ice extent of the whole, north half (>51° N), and south half (<51° N) of the Sea of Okhotsk (Figure S1). For the latitudes of the air parcel and their relative angle variables, we performed backward trajectory analysis to estimate the locations of the air parcel of interest. This method has been widely used for the determination of the source region and the transportation pathway of air pollutants (e.g., Stohl, 1998). We assumed the initial location of this analysis to be at 45° N and 145° E with an altitude of 0 m and calculated the location of the air parcel at hourly intervals till 72 h using wind data. This is not the original observation site but is the most adjacent oceanic location to the original sites where we can avoid the severe effects of geomorphology on the wind direction estimates. The hourly interval wind speeds (west–east and south–north components) at the location of the air parcel were determined by linear interpolation in time and space from JRA‐55 data which provides 3‐hourly interval data at a 1.25° × 1.25° mesh grid. We also did not consider vertical displacement of the air parcel because our observation indicated that the target species flies mostly around the sea surface (lower than altitudes of 50 m).

For the sea ice extent, air parcel latitude, and air parcel angle variables, we performed preliminary analyses to simplify the model structure. That is, for each variable, we included one of the three categories plus the observation duration as the explanatory variables in GLMs and determined the most parsimonious model based on AIC values (Table S1). These preliminary analyses showed that the following three variables would explain the gull observation probabilities more than the others: (1) the sea ice extent of the north half of the Sea of Okhotsk, (2) air parcel latitude 24 h before (hereafter “Lat24”), and (3) air parcel angle 72 h before (hereafter “Deg72”) (Table S1). We next constructed the GLMs with all the combinations of those three variables plus the observation duration and selected the most parsimonious model with the lowest AIC as the best model for the forecast. We conducted these analyses in the R software with packages “lme4” and “MuMin” (Bates et al., 2014). All the explanatory variables were scaled before being analyzed. Based on the above‐mentioned model selection, we employed Lat24, the ordinal date, and the fixed observation time (60 min) as the inputs of the statistical model (see Section 3 for the outcome of the model selection).

#### 2.2.3 Running forecast systems

We attempted one‐day to five‐day forecasts of the observation probability of the focal species for both methods using the output of the global numerical weather forecast provided by the JMA. Although JMA adopted an ensemble weather forecast, in which 51 forecast results are produced with perturbed initial conditions, we used a single forecast with no initial perturbation for simplicity. Method A seeks the node out of the 16 nodes in Figure 3 which exhibits the most similar SLP pattern to one of the focal days in JMA's forecast. The probability of the corresponding node (Figure 3) was assumed to represent the probability of the targeting day. The resemblance was quantified based on the Euclidian distance between the SLP patterns in the node and that forecasted. Thus, we only need the snapshot of the predicted SLP on a focal day. In Method B, a 6‐h interval predicted winds during 24‐h prior to the focal day was used to compute the Lat24 based on the backward trajectory method. To predict the probability of day *X*, 6‐h interval winds from day *X* − 1 through day *X* were used for this computation.

### 2.3 Validation of forecast systems

Commonly, the forecast probability is quantitatively evaluated using the Brier score (Brier, 1950), but the calculation of the score requires many results from the probabilistic forecast. Hence, we conducted two alternative assessments: qualitative analysis and actual observation after the launch of the developed forecast systems.

#### 2.3.1 Qualitative analysis

The proposed methods have been assessed qualitatively for two gull presence days (February 10, 2020 and December 20, 2020) and two absence days (January 2, 2020 and January 9, 2021). Ross's Gull was observed just once (December 20, 2020) in the 2020–2021 winter season, which is not included in the training data of Methods A and B. We thus included this day plus the last presence day of the previous season (February 10, 2020) for the assessment. We also randomly selected the above absence days during the same periods. We evaluated the prediction probabilities for these cases with different lengths of lead time from 1 day through 5‐day prior to the above days and assessed how those values varied according to the lead time and the meteorological reasons behind it.

#### 2.3.2 Observation based on the developed forecast systems

To further evaluate the reliability of the developed forecast systems, we conducted field observation in the 2020–2021 (2 days and one of these is the same used for the qualitative analysis) and 2021–2022 (12 days) winter seasons. These trials were conducted under which the forecast systems provided lower to higher gull observation probabilities.

## 3 RESULTS

### 3.1 Monitoring of Ross's Gull

Ross's Gull was observed in 13 out of 16 monitored winter seasons. The number of days with gull sightings per year (±SD) was 3.50 (±4.35, Range: 0–16). The daily number of gulls observed (±SD) was 26.78 (±54.20, Range: 1–251) and 34.97 (±64.39, Range: 1–251) for the whole (*n* = 60) and final datasets (*n* = 33), respectively. 33.33% (*n* = 20), 13.33% (*n* = 8), and 53.33% (*n* = 32) of the total records (*n* = 60) were obtained in January, February, and December, respectively.

### 3.2 Method A: Forecast based on SLP patterns

The estimated gull observation probability ranged from 0% to 85.7% and showed a strong dependence on the weather patterns (Figure 3). Higher probabilities (44%–85.7%) are found in nodes arranged on the top and right sides of the SOMs (e.g., #003, #004, and #008) (Figure 3). These top‐three nodes represented a developed low‐pressure system located over the Kuril Islands and Kamchatka Peninsula. In these patterns, the observation site was covered by northerly winds prevailing from eastern Sakhalin and the Gulf of Patience, suggesting Ross's Gull has been forced to travel from these areas to the Shiretoko Peninsula due to strong winds. In contrast, the probabilities are relatively low for nodes at the left column or bottom row of the SOMs; the surface wind, especially from the north, around the Shiretoko Peninsula was weak. The bottom right nodes exhibited the westerly wind which is presumably caused by the northward shift of the low‐pressure system.

### 3.3 Method B: Forecast based on a statistical model and backward trajectory

**FIGURE 4:**
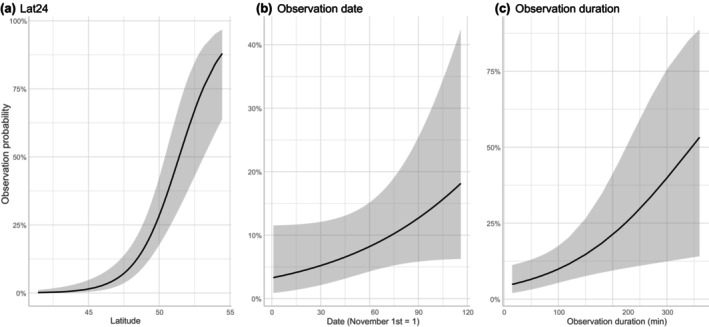
The relationships between observation probabilities and three explanatory variables in the best GLM. (a) Lat24 (the latitudes of the air parcel 24 h before it arrived at the Shiretoko Peninsula), (b) observation date, and (c) observation duration. Shadow areas indicate 95% confidence intervals. For a regression curve of each focal variable, fixed values of the other two variables were used.

The minimum AIC model included Lat24 (the latitudes of the air parcel 24 h before it arrived at the initial location of the backward trajectory analysis), the ordinal date, and the observation duration (Table 1, Figure 4). More specifically, increasing Lat24 (i.e., strong northerly winds) and seasonal progress were estimated to increase observation probabilities of Ross's Gull (Figure 4). The estimated gull observation probability ranged from 0% to 95.5% for a fixed observation duration (60 min) depending on the combinations of the above two variables.

**TABLE 1:** Top 10 models of the model selection in the GLM analysis.

Model no.	Intercept	Date (November 1 = 1)	Deg72	Sea ice extent (north)	Lat24	Duration	df	logLik	AIC	ΔAIC
1	−2.38	0.48			1.74	0.59	4	−52.98	114.00	0.00
2	−2.44	1.22		−0.79	1.75	0.64	5	−52.03	114.10	0.11
3	−2.33				1.76	0.46	3	−54.50	115.00	1.04
4	−2.37	0.48	0.10		1.71	0.59	5	−52.95	115.90	1.94
5	−2.42	1.21	0.10	−0.78	1.72	0.64	6	−52.00	116.00	2.05
6	−2.34			0.24	1.75	0.51	4	−54.06	116.10	2.17
7	−2.31		0.13		1.72	0.47	4	−54.44	116.90	2.94
8	−2.27				1.85		2	−57.00	118.00	4.04
9	−2.32		0.11	0.24	1.72	0.51	5	−54.03	118.00	4.10
10	−2.28	0.26			1.85		3	−56.48	119.00	5.00

*Note*: “Date,” “Deg72,” “Lat24,” and “duration” indicate the ordinal date (November 1st = 1), the air parcel angles 72 h before, the latitudes of the air parcel 24 h before, and the observation duration. Coefficients of included explanatory variables (Date, Deg72, and Sea ice extent) in each model are shown.

### 3.4 Forecast systems and qualitative analysis for validation

**FIGURE 5:**
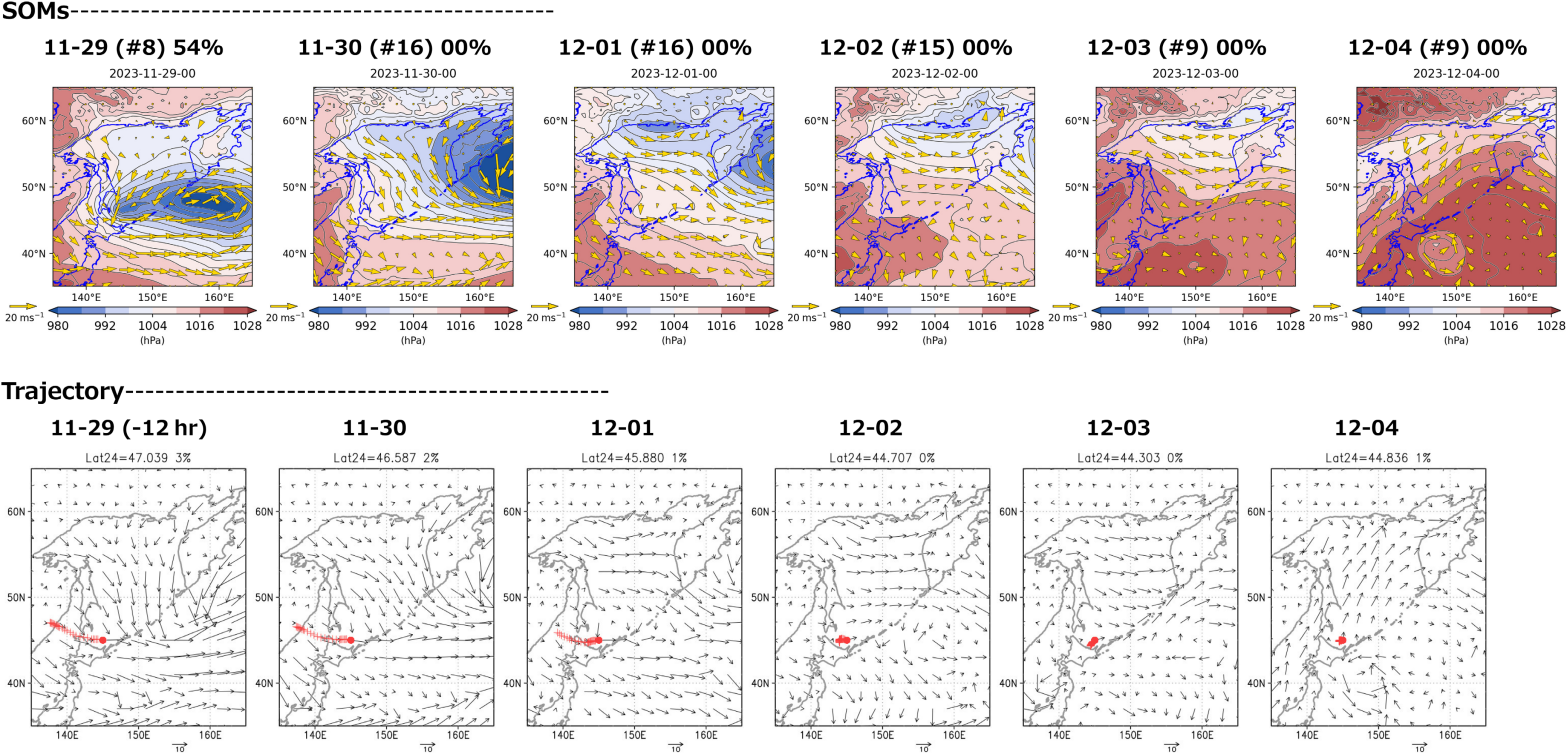
Examples of the forecasted probabilities from SOMs (Method A) and backward trajectory (Method B). Note that the location for calculating Lat24 in Method B (red dots) was slightly different from the original observation sites to avoid the severe effects of geomorphology on the wind direction estimates (see also Section 2).

We combined the above two methods with the global numerical weather forecast and were able to get 1‐ to 5‐day forecasts of the observation probabilities of the focal species for both methods (Figure 5). To validate the reliability of both forecast systems, we then assessed the dynamics of the gull observation probabilities 1–5 days prior to the four cases as follows: The evaluation of the forecasted probability suggests both methods can distinguish sighted and non‐sighted days with higher and lower forecasted probabilities, respectively (Appendix S3). However, their forecast skills were found to be highly dependent on input data precision of the position and strength of the low‐pressure system east of Hokkaido. In the case of January 2, 2020 (non‐sighted day), both methods forecasted a low probability of the result being available 4 days before the focal day. For the sighted days of February 10 and December 20, 2020, both methods also showed higher observation probabilities (Table S2). In a non‐sighted case on January 9, 2021, however, a small low‐pressure system generated off western Hokkaido and passed over the Sea of Okhotsk affected the discrepancy between the two methods. It was found that even small and weak low‐pressure systems, which are likely obscured in the classification of the large‐scale SLP pattern, can be a factor that changes the local wind system. In Method A, the SLP pattern was classified to node #012 in which northerly wind is predominant over the Sea of Okhotsk, and thus, the forecasted probability was relatively high (33.3%) 1–2 days prior (Table S2). In contrast, Method B forecasted low probability (approximately 1%–8%) 5 days prior to the focal day (Table S2). This is presumably because the trajectory analysis in Method B reflects the local wind patterns near the observation site induced by the small low pressure.

### 3.5 Observation based on the forecast systems

2–28 Ross's Gulls were observed on six out of 14 observation days (Table 2). Although Ross's Gull was not always observed on the days when relatively higher probabilities were forecasted by one of the methods (e.g., December 27, 2021, and January 19, 2022), the positive outcome days tended to have higher forecast probabilities during most of the 5‐day forecast periods in both forecast systems. By contrast, unsighted days showed relatively lower forecasted probabilities (<10%) in at least one of the forecast systems throughout the 5‐day forecast period (e.g., December 29, 2021, and January 8, 2022). Overall, these observations demonstrate the developed forecast systems to be effective, especially for the cases in which both systems forecasted higher observation probabilities.

**TABLE 2:** Observation outcomes based on the developed forecast systems.

Observation day	Forecast probability						Outcome
		−5 day	−4 day	−3 day	−2 day	−1 day	
2020.12.20	Method A	85.7	85.7	33.3	85.7	33.3	1 (4)
	Method B	7.38	7.75	8.13	7.57	12.41	
2020.12.31	Method A	33.3	33.3	33.3	85.7	85.7	0
	Method B	1.3	1.4	1.7	2.8	5.3	
2021.11.27	Method A	25.0	25.0	25.0	25.0	27.3	1 (3)
	Method B	2.1	1.8	2.0	47.2	73.4	
2021.11.28	Method A	25.0	25.0	25.0	42.9	25.0	1 (28)
	Method B	22.4	6.3	7.6	26.6	5.8	
2021.12.18	Method A	33.3	33.3	33.3	85.7	85.7	1 (2)
	Method B	42.8	19.2	30.6	22.1	12.3	
2021.12.26	Method A	66.7	85.7	85.7	85.7	85.7	1 (14)
	Method B	0.8	62.3	1.3	1.1	6.5	
2021.12.27	Method A	85.7	33.3	33.3	85.7	85.7	0
	Method B	3.0	1.4	10.9	10.4	10.7	
2021.12.29	Method A	25.0	25.0	42.9	42.9	42.9	0
	Method B	6.3	1.2	1.1	1.8	1.5	
2022.01.05	Method A	85.7	27.3	27.3	27.3	27.3	1 (18)
	Method B	2.1	23.8	45.9	67.7	65.6	
2022.01.08	Method A	27.3	25.0	25.0	27.3	27.3	0
	Method B	2.5	2.6	1.9	1.6	1.8	
2022.01.19	Method A	44.4	44.4	44.4	44.4	44.4	0
	Method B	9.3	31.9	44.1	27.7	40.1	
2022.01.21	Method A	44.4	44.4	44.4	44.4	44.4	0
	Method B	3.2	3.1	3.1	3.2	3.3	
2022.01.22	Method A	25.0	25.0	25.0	27.3	27.3	0
	Method B	2.9	7.6	12.4	12.1	13.8	
2022.01.29	Method A	0.0	33.3	33.3	33.3	33.3	0
	Method B	1.8	2.4	1.8	1.7	1.7	

*Note*: “1” and “0” in the “Outcome” column indicate gulls to be sighted and unsighted, respectively, on each observation day. Values in parentheses indicate the number of gulls. For Method B, we used Lat24, days from November 1st of the season, and 60 min of continuous observation as the model inputs. Forecast probabilities between 1 and 5 days before the observation day are shown for both methods. The red text indicates the highest forecast probabilities of both systems for each day.

## 4 DISCUSSION

The present study proposed two real‐time forecast systems for the observation probabilities of Ross's Gull based on its long‐term data with weather prediction combined. We demonstrated that although the two systems would be complementary in some situations, both were able to forecast dynamic and reasonable observation probabilities of the target species. These forecast systems, therefore, are expected to dramatically increase the probability of detection of this iconic bird for a variety of people, underpinning their better experiences (Gaston, 2022).

Data of not only migratory/vagrant bird species but also many other flying animals (e.g., bats and butterflies) have been accumulated by professional and/or citizen scientists for many years worldwide (Cryan, 2003; Howard & Davis, 2015; Jiguet & Barbet‐Massin, 2013; McGuire & Boyle, 2013; Sanderson et al., 2021), and their movement/dispersal, migration, and vagrancy are suggested to be linked to meteorological patterns and factors (Drake & Farrow, 1988; Sapir et al., 2011; Shamoun‐Baranes et al., 2010). Such meteorological information can also be available at high resolution across space and time globally (e.g., Buontempo et al., 2022). Therefore, our framework can be applied to many different animals across regions.

### 4.1 Accuracy of the proposed forecast systems

The forecast accuracy in Method A depends on both the certainty of surface pressure patterns in the numerical weather prediction model and the judgment of the similarities in pressure patterns between the nodes and those predicted. Since we aimed to assess how the cyclonic/synoptic‐scale meteorological conditions can forecast the observation probabilities of the focal species, we used the SOMs over the western North Pacific including the Sea of Okhotsk. However, our validation suggested that the local‐scale pressure patterns difficult to identify from the SOMs can also play a role in predicting wind direction/strength around the study region (i.e., the case on January 9, 2021). Although Ross's Gull is unlikely to be observed under southerly wind conditions, Method A tended to categorize those days into nodes with higher observation probabilities. Small‐scale low pressures are known to frequently occur around Hokkaido (Tamura & Sato, 2020). Hence, it is a promising next step to modify forecast settings that can represent such small‐scale pressure patterns and wind conditions by, for example, narrowing the focal region/area or increasing segmentation of the SOMs nodes. The latter option may also enable us to identify more specific meteorological patterns with higher observation probabilities. Future work should thus continue to accumulate observation data for such possible improvement.

One of the major predicters incorporated in Method B was backward trajectory based on wind speeds in various horizontal directions. Hence, the forecast accuracy would be linked to the estimated location of the air parcel 24 h before the observation timing (i.e., Lat24) from the trajectory analysis. In this context, forecast probabilities may be unreliable under which low pressures pass around the study region just before the forecast time because small differences in the passage timing would result in larger errors in the Lat24 estimates. The uncertainty of the statistical model estimate may also lower the reliability of forecast probabilities. Although the developed gull forecasts are currently updated every 9 A.M., future trials for subdividing the forecast timing of the day (i.e., updates at multiple times of the day) may help understand the tendency and uncertainty in the Lat24 estimates. Improving statistical model fitting may also be possible by not only initiating backward trajectories at more precise arrival timing of the focal species to the observation points but also considering other biologically relevant factors such as the location of a specific wintering area.

### 4.2 The importance of the Sea of Okhotsk as a wintering area for Ross's Gull

Ross's Gull is thought to stay in Arctic regions year‐round. However, less information about its specific non‐breeding area has been available. Research has revealed that tens of thousands of Ross's Gull are observed at cape Barrow, Alaska, every fall migration season (Divoky et al., 1988; Maftei et al., 2014). Another tracking research has also clarified that some Ross's Gulls overwinter in the sea ice edge of the northern Labrador Sea (Maftei et al., 2015). In addition to these, our monitoring records and analyses showed that strong northerly winds bring a substantial abundance of this species to the southern edge of the Sea of Okhotsk, and the chance of these events can increase with the progress of the winter season, suggesting the northern parts of the sea to be another regular wintering region of Ross's Gull. Indeed, observational research from vessels has documented sightings of this species over the Sea of Okhotsk, especially in open waters around the northeastern part of the sea (Artukhin, 2019). Although the latitude of the Sea of Okhotsk is much lower than that of previously documented wintering grounds of this species and our model showed the sea ice coverage to have little effect on its observation in our specific study site, the sea is known to be covered by seasonal sea ice substantially (Honda et al., 1999) and this species tends to occur at the edge of sea pack ice (Maftei et al., 2015). Hence, stable habitat availability in the region may explain the frequent Ross's Gull occurrences. Nevertheless, species dependent on Arctic ecosystems including Ross's Gull would be most susceptible to expected increasing threats of climate change (Gilg et al., 2012; Moore & Huntington, 2008). Future work should therefore seek to understand the seasonal distribution of this species over the sea, what environmental factors enable them to overwinter, and how ongoing climate changes will affect the seasonal occurrences of the species.

### 4.3 Limitation

The developed forecast framework has some limitations that should be improved in future work. First, the two forecast methods provided different forecast probabilities in some cases. This is likely because the arrival of this species to the observation sites would be related to both relatively local (i.e., northerly wind strength) and large (i.e., surface pressure patterns around the Sea of Okhotsk) scale meteorological factors. These two forecasts are therefore complementary to knowing a more reliable observation probability, and further improvement may be possible by jointing these into a single forecast system. Second, although our observation data showed the observed gull abundance/occurrence would vary between years greatly, both methods were unable to forecast such yearly variation for the coming year, highlighting the importance of clarifying its drivers for improving the forecast skills. Third, we used a single forecasted data with no perturbation applied to the initial conditions to calculate the daily observation probabilities of our target species in the two forecast methods. Although the two developed forecast systems of our target species seem reliable in their current settings, both cannot consider the uncertainties of some meteorological events such as the presence/absence and passage of small cyclones over the study region, and these uncertainties may bias the forecasted observation probabilities. It is a promising next step to evaluating how such uncertainties can be improved by considering various members of the ensemble forecasting with different perturbations. Finally, the observer's better experiences may depend not on the presence of the gull but on its abundance. It is therefore key to develop a forecast system based on the abundance in future work.

## 5 CONCLUSION

Regardless of the above possible limitations of the proposed forecast systems, the present study proves an interdisciplinary approach between ecology and meteorology to be promising in acquiring a deeper insight into the causes of migration/vagrancy of a specific bird species and the practical application for bird observation forecasts. Our model species was more challenging to apply the developed approaches because this species may be nomad rather than doing seasonal migration, and thereby may show great inter‐annual variations in occurrence or abundance in our study site. However, our approaches may be easier to apply for species that show regular annual migrations, such as hawks, waterbirds, and many insectivore passerines, because their migration timing would be tightly linked to fixed annual meteorological cycles and conditions. Although which forecast method is more cost‐effective may depend on the data availability of target species, the species‐specific movement patterns, and the associated important meteorological factors, our study sheds light on a previously overlooked potential of existing biological monitoring data to facilitate not only a better understanding of animal migration/vagrancy but also the personal experiences of nature lovers with diverse interests.

## AUTHOR CONTRIBUTIONS

**Masayuki Senzaki:** Conceptualization (lead); data curation (equal); formal analysis (equal); funding acquisition (lead); investigation (supporting); methodology (equal); project administration (lead); resources (lead); supervision (lead); validation (equal); visualization (equal); writing – original draft (lead); writing – review and editing (equal). **Kenta Tamura:** Conceptualization (equal); data curation (equal); formal analysis (equal); methodology (equal); project administration (equal); resources (equal); software (equal); validation (equal); visualization (equal); writing – original draft (supporting); writing – review and editing (equal). **Yoshiaki Watanabe:** Conceptualization (equal); data curation (lead); investigation (lead); project administration (equal); validation (equal); writing – review and editing (supporting). **Megumi Watanabe:** Conceptualization (equal); data curation (equal); investigation (equal); project administration (equal); writing – review and editing (supporting). **Tomonori Sato:** Conceptualization (equal); formal analysis (lead); funding acquisition (equal); methodology (equal); project administration (equal); resources (equal); software (equal); supervision (equal); validation (equal); visualization (equal); writing – original draft (supporting); writing – review and editing (supporting).

## CONFLICT OF INTEREST STATEMENT

We have no competing interests.

## Supporting information


Appendix S1.

## Data Availability

The dataset supporting this article is available in Supporting Information. JRA‐55 is openly available via the internet (https://jra.kishou.go.jp/JRA‐55/index_en.html). Numerical weather forecast data are distributed by Research Institute for Sustainable Humanosphere, Kyoto University (http://database.rish.kyoto‐u.ac.jp/index‐e.html). The OISST dataset is available on the NOAA website (https://www.ncei.noaa.gov/products/optimum‐interpolation‐sst). All codes used for analyses of Method A are developed by the authors and available upon reasonable request. R code used in Method B is available in Supporting Information. Non‐profit users can access a webpage of the real‐time forecasts provided by the developed systems upon request to the authors.
