# Supplementary material for: Rare bird forecast: A combined approach using a long‐term dataset of an Arctic seabird and a numerical weather prediction model
Source: Ecol Evol. 2024 Jun 25;14(6):e11388. doi: 10.1002/ece3.11388 (PMC11199128; doi:10.1002/ece3.11388)
Supplement: Supplementary file 1 — Appendix S1. [file ECE3-14-e11388-s001.zip › SupplementFinal_2nd.docx]

**Supporting Information**

**Appendix S1:** The sea ice extent of the north half (>51° N) and south half (<51° N) of the Sea of Okhotsk.


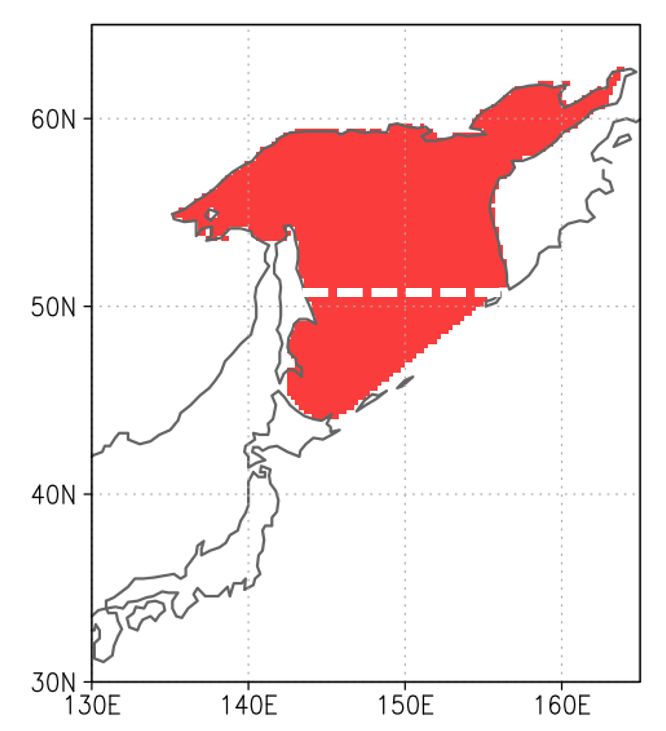


Figure S1. The area of the Sea of Okhotsk for the sea ice extent calculation. The red area indicates the whole area. The white dashed line is a boundary between the north and south areas.

**Appendix S2: Results of the model selection for Method B.**

Table S1. The results of the model selection for the sea ice extent, the air parcel latitude, and the air parcel angle variables. We used the observation duration (minutes) as an additional factor in each model. The lowest AIC was indicated in bold.

| **Response variable** | **Focal explanatory variable** | **AIC** |
| --- | --- | --- |
| Presence/absence | Sea ice extent (whole) | 159.88 |
|  | **Sea ice extent (north)** | **159.50** |
|  | Sea ice extent (south) | 161.01 |
| Presence/absence | **Lat24** | **114.99** |
|  | Lat48 | 122.43 |
|  | Lat72 | 127.06 |
| Presence/absence | Deg24 | 160.62 |
|  | Deg48 | 160.96 |
|  | **Deg72** | **159.82** |

**Appendix S3: Full descriptions for the validation of the four representative cases.**

**1) 10 Feb 2020 (Abundance=150)**

Shiretoko area was covered by the northerly to north-northwesterly wind driven by strong low pressure centered on the south of Kamchatka Peninsula and the weak small low-pressure system on the east of Shiretoko. The location and intensity of the strong low have been well predicted at least three days before the target day. The backward trajectory showed that the pathway of the air parcel reached Shiretoko had traveled from southern Sakhalin or Russian Far East. The Lat24 was greater than 47degN for predictions with a lead time longer than 4 days, and the predicted probability stably was around 20% or more after 7^th^ February (i.e., 3-day at the lead time) (Table S2). The weather pattern of the target day (10^th^ February) has been categorized as node #07 or #03 (Table S2). As a result, the probability from SOM-based prediction remains 27.3% or 44.4% since the five-day-long prediction is available on 5^th^ February (Table S2).

**2) 20 Dec 2020 (Abundance=4)**

This event is characterized by the developed low pressure centered on the southeast of the Kamchatka Peninsula. The dominant wind around Shiretoko was strong and its direction was northwesterly to north-northwesterly. The location and intensity of the low pressure were predicted well by JMA’s model at least five-day before the target day, and the prediction was stable thereafter. The predictions performed close to the target day (1–3-day lead time) showed a small-scale cyclonic system around 45N, 153E, but it did not affect local surface wind near Shiretoko. The backward trajectory showed a route from Russian Far East on 5-day before the event and passed through the Soya Strait, reaching Shiretoko. The predicted Lat24 ranged from 47.6N to 48.5N, and the probability of 1-day was 12.4 % (Table S2). The weather patterns of the target day predicted with a 5-day lead time or shorter all correspond to the SOM’s node #08 or #12, leading to the predicted probability to be 33.3 or 85.7 % (Table S2). This means in this case we were able to predict the gull presence in advance of 5 days, and the prediction was reliable.

**3) 02 Jan 2020 (Abundance=0)**

On January 2, 2020, the low-pressure center was located in the northern part of the Sea of Okhotsk, and the Shiretoko area was covered by west-northwesterly winds. The position of this low pressure varies among predictions, showing a slight northward shift of the center position as shortening the lead time (i.e., as approaching the target day). The backward trajectory shows the pathways originated from Russian Far East, and the trajectory travels across the northern fringe of Hokkaido Island. The results of Lat24 were approximately 46N throughout the 5 days in advance to the target day (Table S2). This result leads to the low probability (<4%) since 28 December, indicating that the low probability for this case was predictable at least five days before the target day. The weather pattern obtained 5 days before (i.e., 28 December) was initially categorized as the SOM’s node #04 which represents a high presence probability of the focal species (66.7%) (Table S2). As approaching the target day, however, the predicted probability dropped clearly and became 0% in the predictions obtained on 29 December and thereafter (Figure S2). This improvement in the prediction probably reflects the updated forecast in which there was a slight northward shift of the cyclone position.

**4) 09 Jan 2021 (Abundance=0)**

The synoptic weather pattern represents the strong low pressure around the Kamchatka Peninsula, and the low-pressure area extends toward Hokkaido Island forming two local low areas in the northern and southern parts of the Sea of Okhotsk. Since the southern low area is located close to Hokkaido, its position will impact the local winds around the monitoring site. The backward trajectory ended its tracking when the air parcel travels over Hokkaido Island, resulting in low Lat24 and low probability (approximately 1–8%) for this case (Table S2). On the other hand, the weather pattern classification based on SOMs tended to recognize the low around the Kamchatka Peninsula as a major system in the domain. Thus, the classification did not account for the presence of the small-scale low near Shiretoko, and the pattern was judged to belong to the nodes whose probability is high. As a result, the probability was predicted to be relatively high (20–33.3%) despite the gull species was not observed in this case.

Table S2. Forecast results of the four representative cases for validation.

| **Focal date** | **Forecast date** | **Node** | **SOMs (%)** | **Lat24** | **Days from 11/1** | **Trajectory (%)** | **Outcome** |
| --- | --- | --- | --- | --- | --- | --- | --- |
| 2020/1/2 | -1 | 14 | 0 | 46.46 | 62 | 4.52 | 0 |
|  | -2 | 14 | 0 | 45.93 | 62 | 3.24 | 0 |
|  | -3 | 14 | 0 | 46.01 | 62 | 3.41 | 0 |
|  | -4 | 13 | 0 | 45.97 | 62 | 3.32 | 0 |
|  | -5 | 4 | 66.7 | 45.97 | 62 | 3.32 | 0 |
| 2020/2/10 | -1 | 3 | 44.4 | 49.13 | 101 | 33.78 | 1 (150) |
|  | -2 | 3 | 44.4 | 48 | 101 | 19.60 | 1 (150) |
|  | -3 | 3 | 44.4 | 48.21 | 101 | 21.85 | 1 (150) |
|  | -4 | 3 | 44.4 | 47.17 | 101 | 12.41 | 1 (150) |
|  | -5 | 7 | 27.27 | 45.07 | 101 | 3.47 | 1 (150) |
| 2020/12/20 | -1 | 12 | 33.3 | 48.46 | 49 | 12.41 | 1 (4) |
|  | -2 | 8 | 85.7 | 47.62 | 49 | 7.57 | 1 (4) |
|  | -3 | 12 | 33.3 | 47.74 | 49 | 8.13 | 1 (4) |
|  | -4 | 8 | 85.7 | 47.66 | 49 | 7.75 | 1 (4) |
|  | -5 | 8 | 85.7 | 47.58 | 49 | 7.38 | 1 (4) |
| 2021/1/9 | -1 | 12 | 33.3 | 47.21 | 69 | 7.97 | 0 |
|  | -2 | 12 | 33.3 | 44.63 | 69 | 1.58 | 0 |
|  | -3 | 11 | 20 | 44.07 | 69 | 1.10 | 0 |
|  | -4 | 12 | 33.3 | 43.7 | 69 | 0.87 | 0 |
|  | -5 | 11 | 20 | 44.1 | 69 | 1.12 | 0 |
